# X-Ray Induced DNA Damage and Repair in Germ Cells of *PARP1^−/−^* Male Mice

**DOI:** 10.3390/ijms140918078

**Published:** 2013-09-04

**Authors:** Paola Villani, Anna Maria Fresegna, Roberto Ranaldi, Patrizia Eleuteri, Lorena Paris, Francesca Pacchierotti, Eugenia Cordelli

**Affiliations:** 1 Unit of Radiation Biology and Human Health, Italian National Agency for New Technologies, Energy and Sustainable Economic Development (ENEA), CR Casaccia, Via Anguillarese 301, Roma 00123, Italy; E-Mails: a.fresegna@inail.it (A.M.F.); robran67@hotmail.com (R.R.); patrizia.eleuteri@enea.it (P.E.); lorena.paris@enea.it (L.P.); francesca.pacchierotti@enea.it (F.P.); eugenia.cordelli@enea.it (E.C.); 2 Department of Ecology and Biology, University of Tuscia, Viterbo 01100, Italy

**Keywords:** poly(ADP-ribose)polymerase-1, DNA repair, male mouse germ cells, comet assay, H2AX phosphorylation, ionizing radiation

## Abstract

Poly(ADP-ribose)polymerase-1 (PARP1) is a nuclear protein implicated in DNA repair, recombination, replication, and chromatin remodeling. The aim of this study was to evaluate possible differences between *PARP1^−^^/^^−^* and wild-type mice regarding induction and repair of DNA lesions in irradiated male germ cells. Comet assay was applied to detect DNA damage in testicular cells immediately, and two hours after 4 Gy X-ray irradiation. A similar level of spontaneous and radiation-induced DNA damage was observed in *PARP1^−^^/^^−^* and wild-type mice. Conversely, two hours after irradiation, a significant level of residual damage was observed in *PARP1^−^^/^^−^* cells only. This finding was particularly evident in round spermatids. To evaluate if PARP1 had also a role in the dynamics of H2AX phosphorylation in round spermatids, in which γ-H2AX foci had been shown to persist after completion of DNA repair, we carried out a parallel analysis of γ-H2AX foci at 0.5, 2, and 48 h after irradiation in wild-type and *PARP1^−^^/^^−^* mice. No evidence was obtained of an effect of PARP1 depletion on H2AX phosphorylation induction and removal. Our results suggest that, in round spermatids, under the tested experimental conditions, PARP1 has a role in radiation-induced DNA damage repair rather than in long-term chromatin modifications signaled by phosphorylated H2AX.

## 1. Introduction

The maintenance of DNA integrity in the paternal genome is of utmost importance for reproduction and it is well known that DNA lesions in germ cells can be transmitted to the next generation [1]. Germ cells need efficient systems to repair DNA damage to prevent inheritable mutations. Differences have been observed among DNA repair pathways in somatic and male germ cells, in which the expression or the presence of some DNA repair proteins depends on the phase of germ cell development [2–9].

Poly(ADP-ribose)polymerase-1 (PARP1) is one of the members of a family of nuclear proteins involved in several cellular processes like DNA repair, replication and transcription, chromatin structure, and intracellular calcium signaling, and is critical for the long-term maintenance of genomic stability [10]. Activation of PARP1 is one of the immediate responses of eukaryotic cells to DNA damage. It recognizes DNA strand breaks and, at the site of breakage, catalyzes the transfer of the ADP-ribose moiety from the respiratory co-enzyme NAD+ to nuclear protein acceptors [11,12]. PARP1 signals the presence of DNA lesions to downstream effectors involved in coordinating the cellular response to DNA damage, recruits repair enzymes to the damaged sites, and affects chromatin structure to allow repair factors to access DNA lesions [11,13–15]. PARP1 has been involved in the repair of various types of DNA lesions by multiple pathways [15]. The role of this enzyme and other PARP family members in DNA repair processes has been extensively studied in somatic cells, both using chemical inhibitors and mouse and cellular models genetically defective for the enzymes [13,16–24]. These studies show that inhibition or lack of PARP slows down DNA repair and increase the cytotoxicity of ionizing radiation and alkylating agents. The contribution of PARP1 to DNA repair in male germ cells has been considerably less investigated. It is known that germinal cells are characterized by a high expression level of PARP [25] and difference among testis cell subpopulations in PARP activity and production of ADP-ribose polymers was also observed [26–30]. In male germ cells poly(ADP ribose) metabolism is important for the regulation of meiotic process and seems to have a role in DNA repair and chromatin remodeling in post-meiotic cells [27,28,30–32]. A delay of radiation-induced DNA damage repair was shown in cultured rat spermatocytes and spermatids [25], and mouse spermatids *in vivo* [3] when poly(ADP ribose) metabolism was inhibited by chemical treatment. These studies left unanswered the question of the specific role of PARP1 in the germ cell DNA damage response because of the poor specificity of chemical inhibitors towards different PARP family members [25].

*PARP1* knockout mice have been produced to investigate the specific role of PARP1 in cellular processes [24]. *PARP1^−/−^* mice are more sensitive to the lethal effects of alkylating agents and ionizing radiation, show an increased frequency of spontaneous sister chromatid exchanges in bone marrow cells and increased levels of chromatid and chromosome aberrations after exposure to genotoxic agents [24,33–35]. Although their fertility is not compromised, more subtle effects on the germ cell DNA damage response in these mice cannot be excluded. To our knowledge, no studies have been published so far to evaluate male germ cell capability of *PARP1^−/−^* mice to repair induced DNA damage.

In this study, alkaline comet assay has been applied to evaluate the level of basal and X-ray induced DNA lesions in testis cells from wild-type (WT) and *PARP1^−/−^* mice. In addition, to investigate the role of PARP1 in DNA repair in male germ cells, DNA damage was assessed at different times after irradiation. Exploiting the capacity of comet assay to identify DNA lesions in individual cells *versus* their ploidy [9,36], we evaluated the response to irradiation of different testis cell subpopulations. Post-meiotic early spermatids were the most affected by lack of PARP1. So, in these cells the induction of double strand breaks (DSB) was also specifically investigated by γ-H2AX immunolabeling. Finally, the persistence of γ-H2AX foci after DNA repair was evaluated to assess the role of PARP1 in long-lasting chromatin remodeling [37].

## 2. Results and Discussion

Cytotoxic effects induced by 4 Gy X-rays on WT and *PARP1^−/−^* testis cells were assessed by flow cytometric DNA content analysis 48 h after irradiation. The most radiosensitive testicular cell population is that of differentiating spermatogonia. Thus, shortly after irradiation, cytotoxic effects are reflected by a decrease of the S-phase flow cytometric compartment, which includes mainly proliferating spermatogonia. X-rays induced a comparable cytotoxic effect in WT and *PARP1^−/−^* mice (Table 1), the magnitude of which was in agreement with a previously published dose-effect relationship [38].

Short term genotoxic damage induced by 4 Gy X-rays on WT and *PARP1^−/−^* testis cells was assessed by alkaline comet assay immediately after irradiation; removal of DNA lesions was assessed by comet analyses at 2 and 48 h after treatment. Overall data are summarized in Table 2.

**Figure 1:**
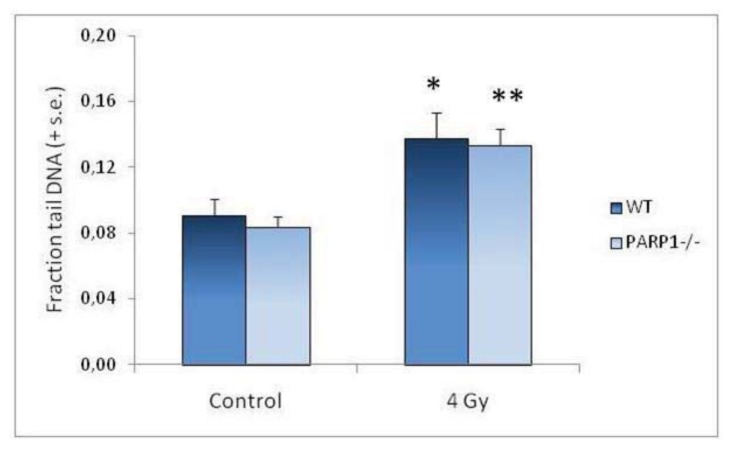
Fraction tail DNA of unirradiated testis cells and of 4 Gy-irradiated cells sampled immediately after exposure. Columns represent the mean of fraction tail DNA values (+ standard error) for each experimental group. Asterisks evidence results significantly different from matched controls (* *p* < 0.05; ** *p* < 0.01).

The mean fraction tail DNA values in control and irradiated testis cells evaluated in WT and PARP1*^−^*^/^*^−^* mice immediately after irradiation are also reported in Figure 1. No differences in the basal level of DNA strand breaks were observed; 4 Gy X-rays induced a similar significant increase of mean fraction tail DNA values in WT and *PARP1^−/−^* mice. These results suggest that, in male germ cells, lack of PARP1 does not affect the level of endogenous damage or modify chromatin structure in a way that makes it more susceptible to radiation-induced lesions.

**Figure 2:**
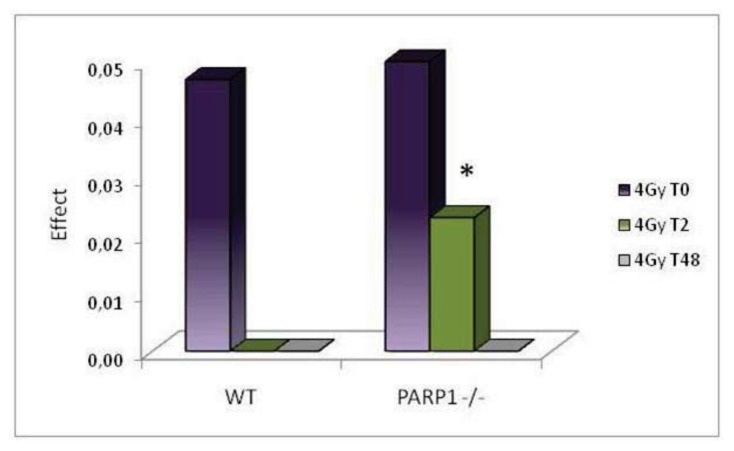
Differences between the mean fraction tail DNA of irradiated and unirradiated groups (Effect) immediately, 2 and 48 h after irradiation in testis cells of wild-type and *PARP1^−/−^* mice. Columns represent the mean values for each experimental group. The asterisk evidences results significantly different from matched controls (* *p* < 0.05).

Histograms in Figure 2 report data on the level of residual damage observed at different times after irradiation expressed as differences between the mean fraction tail DNA of irradiated and unirradiated groups. WT mice showed a complete repair of DNA lesions within two hours after treatment; on the contrary, in *PARP1^−/−^* mice, although the level of damage decreased with time after irradiation, a statistically significant (*p* < 0.05) level of residual damage was still detected two hours after treatment. Similarly to WT animals, 48 h after irradiation no residual damage was detectable in *PARP1^−/−^* mice. These results are in agreement with previous studies in rodent testis cells reporting a significant delay of DNA repair following oxidative stress induced by chemical and physical agents when PARP activity was inhibited by chemical inhibitors [3,25,27], and suggest that PARP1 is specifically involved in DNA strand break repair after X-ray irradiation in one or more spermatogenic cell stages. The complete recovery observed 48 h after treatment indicates that, eventually, the absence of PARP1 is compensated by other proteins with a similar function and/or alternative repair pathways. Previous *in vitro* studies on PARP1 null cells had already suggested that lack of PARP1 decreases the efficiency of and delays DNA repair, although such effects are ultimately overcome by backup mechanisms [19,24,39].

**Figure 3:**
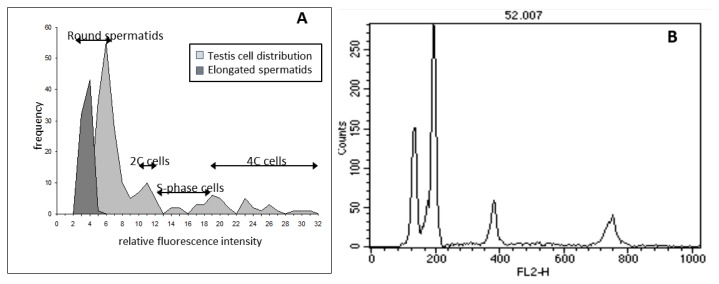
Comparison between DNA content histograms obtained by comet assay (**A**) and flow cytometry (**B**). The histograms were obtained by analyzing the samples of untreated testis cells. Two hundreds and 10,000 cells were analyzed by comet assay and flow cytometry respectively.

In order to investigate the response to the insult of different testis cell subpopulations we exploited the capacity of comet assay to identify DNA lesions in individual cells *versus* their ploidy. The cell distribution obtained by this approach (Figure 3A), that had been previously applied to evaluate DNA damage in different cell cycle phases [40] and different testicular cell types [36], was validated by comparing it with the DNA content distribution histogram obtained by flow cytometry (Figure 3B). The two distributions resulted very similar also considering that flow cytometric histogram was obtained measuring 10,000 cells, while the comet assay histogram is based on 200 cells.

**Figure 4:**
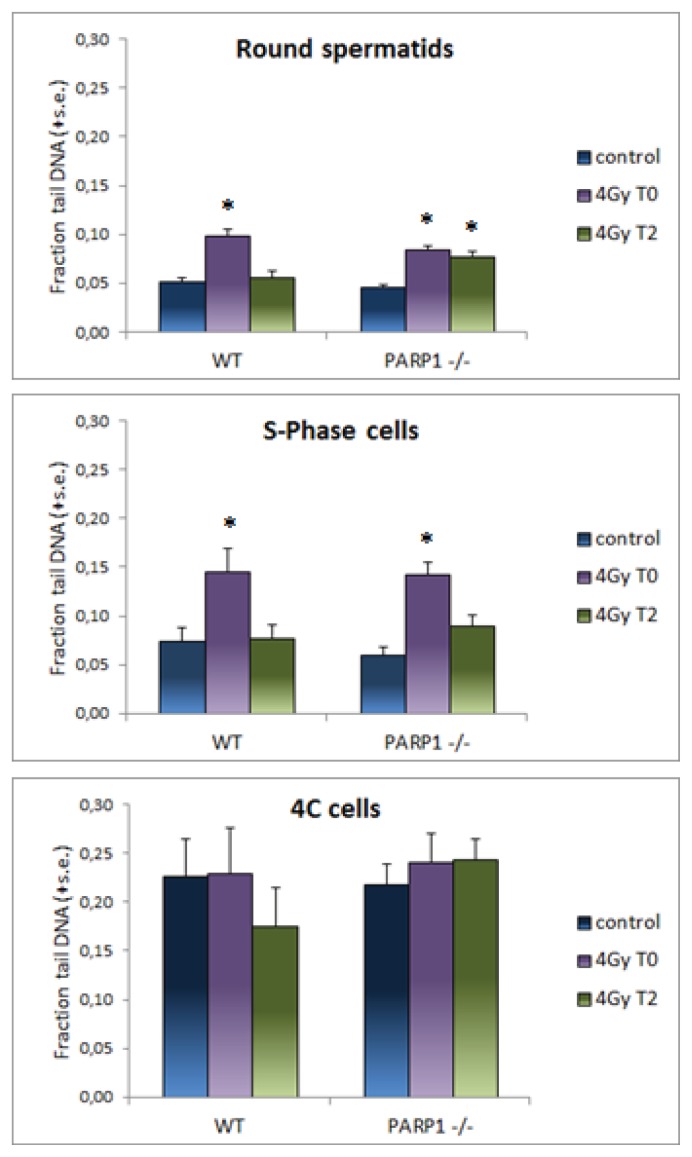
Fraction tail DNA in different testis subpopulations, discriminated on the basis of their ploidy, after irradiation of WT and *PARP1^−/−^* mice. Mean of fraction tail DNA values (+ standard error) are shown for each experimental group. Control, untreated sample; 4 Gy T0, immediately after irradiation; 4 Gy T2, 2 h after irradiation. Asterisks evidence results significantly different from matched controls (* *p* < 0.05).

The mean fraction tail DNA values were then evaluated on cells attributed to different cell subpopulations, as described in the Experimental Section and are reported in Figure 4. The distribution of strand breaks in different cell types of unirradiated testes was similar in WT and *PARP1^−/−^* cells, with four-times higher mean fraction tail DNA values in untreated 4C cells than in the other cell types. This finding could be ascribed to DNA strand breaks generated during meiotic synapsis and recombination occurring in pachytene cells [41,42], which represent a relevant fraction of this subpopulation. Immediately after irradiation an increase of fraction tail DNA values was induced in round spermatids, 2C and S-phase cells while no effect was observed in 4C cells, indicating different sensitivity of various cell subpopulations. This result, in agreement with Zheng and Olive [9], could reflect differences in chromatin condensation and interaction of target DNA with histone and non-histone proteins during the differentiation process that make it differentially susceptible to radiation-induced lesions. It is known that radiosensitivity of testicular subpopulations varies, the mitotically active spermatogonia being the most sensitive to ionizing radiation, and spermatocytes and spermatids the most resistant cells [43]. Two hours after treatment, a significant reduction of radiation damage was observed in all cell subpopulations with the exception of *PARP1^−/−^* round spermatids, indicating that the delay in strand break rejoining observed in testis cells was mainly imputable to this cell subpopulation. This finding is consistent with the observation that round spermatids respond to genotoxic stress with a more elevated production of poly(ADPribose) than other testis cells and provides evidence that lack of PARP1, among all family members, is specifically implicated in the delay of DNA repair shown in rodent post-meiotic cells [3,25] treated with chemical inhibitors of poly(ADP) ribosylation. Considering that ADP-ribosylation of nuclear proteins releases chromatin structure and, in this way, facilitates repair of damaged regions [11,17], it is conceivable that, due to the progressive chromatin condensation occurring during post-meiotic maturation, spermatids increasingly rely upon PARP1 activity to give access to repair enzymes. It was not possible to specifically investigate the requirement for PARP1 in elongated spermatids because our experimental conditions failed to decondense their compact chromatin. However, since all DNA repair processes decrease with maturation, the role of PARP1 in mature spermatids is probably less relevant.

We had previously shown that round spermatids are peculiar also regarding the dynamics of H2AX phosphorylation, which persists long time after DNA damage induced by irradiation has been repaired [37]. To evaluate if PARP1 had also a role in the dynamics of H2AX phosphorylation in these cells, the induction and removal of γ-H2AX foci were compared in round spermatids from *PARP1^−/−^* and WT mice. H2AX phosphorylation has become a popular marker of radiation-induced DSB [44] and more recently, it has been proposed to also mark chromatin modifications independently from the presence of DNA breaks [45–47]. In unirradiated testes, H2AX is highly phosphorylated in the sex body of pachytene cells, which corresponds to the heterochromatic domain of sex chromosomes [41,48] and γ-H2AX marks sites of recombinational DSB preceding chromosome synapsis [41]. After irradiation, increased phosphorylation was found in the whole testis [49] and γ-H2AX foci were detected in A spermatogonia, pachytene spermatocytes, and round spermatids [3,37,42], as well as in neonatal male germ cells [50]. Due to the interplay between Ataxia Telangiectasia Mutated (ATM) kinase protein and poly(ADP-ribosyl)ation that is important for the phosphorylation of H2AX [51] a difference in the basal and radio-induced level of γ-H2AX foci could be expected between *PARP1^−/−^* and WT mice.

**Figure 5:**
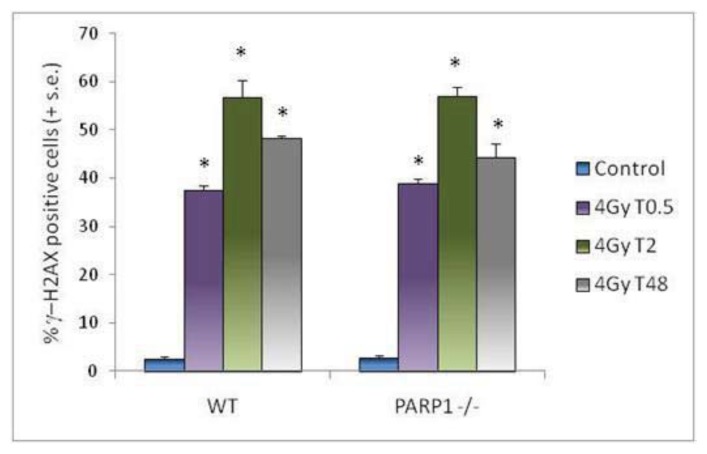
Percentages of γ-H2AX-positive round spermatids. Control, untreated sample; 4 Gy T0.5, 30 min after irradiation; 4 Gy T2, 2 h after irradiation; 4 Gy T48, 48 h after irradiation. Columns represent the mean values (+ standard error) for each experimental group. Asterisks evidence results significantly different from matched controls (* *p* < 0.001).

**Figure 6:**
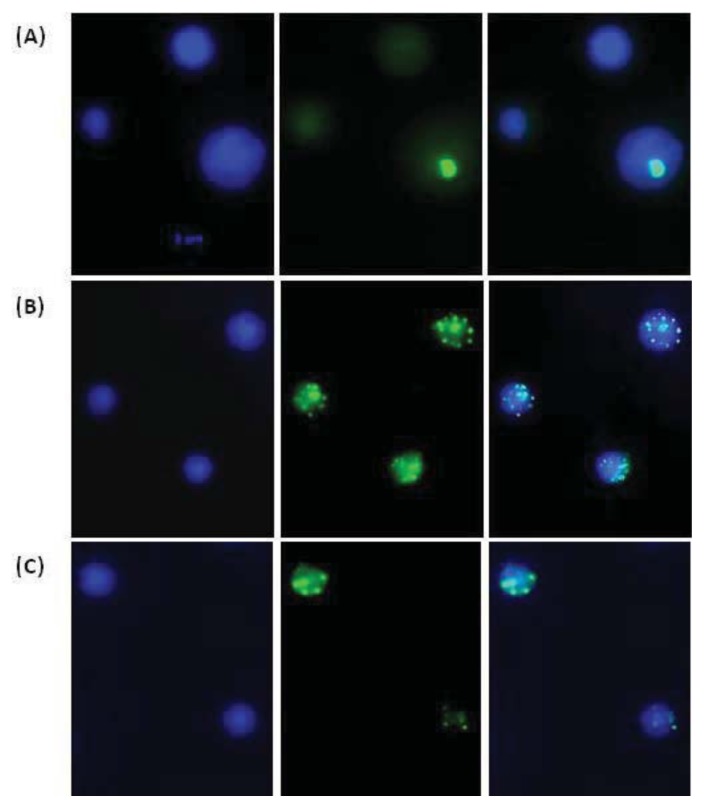
Representative images of γ-H2AX immunostained testicular cells. Left: DNA fluorescence; middle: γ-H2AX fluorescence; right: merge. (**A**) Unirradiated cells: one pachytene spermatocyte showing a bright stained XY body and two round spermatids; (**B**) Three round spermatids 30 min after 4 Gy X-ray; (**C**) Two round spermatids 48 h after 4 Gy X-ray.

Under our experimental conditions, no difference was observed between *PARP1^−/−^* and WT mice in the background frequency of γ-H2AX-positive spermatids (Figure 5). Thirty minutes after irradiation a comparable highly significant increase (*p* < 0.001) of γ-H2AX-positive cells was found in both lines. The subsequent temporal evolution of γ-H2AX foci was also quantitatively similar in WT and *PARP1^−/−^* mice: 2 h after irradiation, a further increase of positive cells was observed, and 48 h after irradiation, although the number of foci was reduced, a high percentage of positive cells was still present. At the same time, the size of foci increased and they became discrete and countable. The average number was 5.7 ± 0.28 in WT and 5.0 ± 0.13 in *PARP1^−/−^* γ-H2AX positive cells, not showing significant differences between the two mouse lines. Such large foci were never detected in the few γ-H2AX positive unirradiated cells. Examples of γ-H2AX labeled testicular cells are shown in Figure 6. A number reduction and a size increase of foci had been observed in somatic and germ cells with time after irradiation and related to clustering of small foci and relocalization of repair enzymes to sites of complex unrepaired DNA lesions or to remaining scaffold structures used for DSB repair [37,52–54]. Our results suggest that PARP1 does not strongly influence the dynamics of H2AH phosphorylation in round spermatids. This finding is not completely consistent with data reported by Ahmed and co-workers [3] who showed that, after a comparable induction of foci by 1 Gy gamma-rays in mice treated or not with PARP chemical inhibitors, significantly more foci remained in PARP inhibited mice at eight hours after irradiation. Such different observations might be explained considering differences in tested doses (1 *vs*. 4 Gy), sampling times (8 h *vs*. 48 h), and analyzed endpoints (number of foci *vs*. percentage of positive cells), or might be actually due to a secondary role of PARP1 among PARP family members in radiation-induced γ-H2AX foci removal, or to a combination of all these factors. Additionally, it should be noted that inhibition of PARP1 is not equivalent to genetic deletion of PARP1, as also suggested by a comparison between RNAi depleted HeLa cells and the same cells treated with a PARP1 inhibitor [55]. In this study, the effects of inhibition were more serious as the PARP1 protein was still able to engage in the formation of a DNA damage chromatin complex making the shift to an alternative repair mode more difficult. Further experiments in *PARP1^−/−^* mice with lower X-ray doses might contribute to solve these issues.

## 3. Experimental Section

### 3.1. Mice

Male C57Bl mice aged 12–14 weeks were obtained from Harlan (Udine, Italy); *PARP1^−/−^* mice were bred at ENEA from founders received from P. de Boer (Nijmegen, The Netherlands) upon licence of G. de Murcia (Strasbourg, France). Animals were maintained under standard conditions (20–22 °C, 60% relative humidity, on a 12 h light/dark cycle, with chlorinated water and feed *ad libitum*). All experimental protocols were reviewed and approved by the Institutional Animal Care and Use Committee.

### 3.2. X-Ray Irradiation

Mice were anaesthetized with Avertin (Sigma Aldrich St. Louis, MO, USA) diluted at 2.5% in sterile saline solution given intraperitoneally at a dose of 10 μL/g b.w. and only the testes region was irradiated with 4 Gy X-rays, the remainder of the body was shielded by 1-mm thick lead shield. Irradiation was carried out with a Gilardoni X-ray machine (15 mA, 250 kV; dose-rate 0.96 Gy/min). At different times after irradiation, mice were sacrificed and testes were removed and minced to obtain cellular suspensions to be analyzed.

### 3.3. Comet Assay of Testis Cells

Alkaline comet assay (pH > 13) was performed on testis cells from control mice and mice irradiated with 4 Gy and sacrificed immediately, 2 or 48 h after irradiation. At least four wild type (WT) and four *PARP1^−/−^* mice were used per experimental point. One testis from each mouse was minced and the cell suspensions were filtered through a 70 μm nylon mesh, centrifuged, resuspended in PBS (approximately 10^7^ cells/mL), and mixed with low-melting point agarose (Bio-Rad, Hercules, CA, USA) to prepare slides for comet assay.

Alkaline comet assay was performed as described by Singh *et al*. [56] with minor modification [36]. Immediately before scoring, slides were stained with 12 μg/mL ethidium bromide (Sigma-Aldrich, St. Louis, MO, USA) and examined, at 200× magnification, with an Olympus fluorescence microscope. Slides were analyzed by a computerized image analysis system (Delta Sistemi, Rome, Italy). To evaluate the amount of DNA damage, computer generated fraction tail DNA values were used. Two-hundred cells were scored for each mouse from two different slides. Elongated spermatids, morphologically recognized, were not included in DNA damage analysis because our standard conditions failed to decondense their compact chromatin. Furthermore, the integral fluorescence intensity of each comet was taken as a measure of DNA content and used to discriminate cells with different DNA content [40]. The procedure to assign cells to the various subpopulations was adapted from the method previously described by Zheng and Olive [9]. In particular, cells were classified as haploid spermatids on the basis of DNA content distribution histograms and morphology; this subpopulation is defined “round spermatids” in the manuscript, although it is rather broad and includes different maturation stages from early round spermatids up to the elongating ones. Somatic and germinal cells, were considered 2C if their total fluorescence was comprised between one standard deviation minus and one standard deviation plus twice the mean haploid cell value. Cells with total fluorescence higher than double fluorescence of 2C cells were considered 4C. Cells included between 2C and 4C populations were considered S-phase cells. A representative histogram of relative fluorescence intensity obtained from a control sample is reported in Figure 3A and compared with the DNA distribution histogram obtained by flow cytometry on the same sample (Figure 3B).

### 3.4. Immunofluorescent Analysis of H2AX Histone Phosphorylation

Immunofluorescent analysis of H2AX histone phosphorylation was performed on round spermatids in groups of 4 *PARP1^−/−^* and 4 WT mice irradiated with 4 Gy X-rays and sacrificed 30 min, 2 h or 48 h later. Four unirradiated mice of each line were used as matched controls. Testis cell suspensions in PBS were mechanically prepared and filtered through 70 μm nylon mesh. The suspensions were fixed in 70% ethanol and diluted to a concentration of approximately 4 × 10^5^ total cells/mL. Cells were cytospun onto slides (10 min, 600 rpm), air-dried, and re-fixed for 30 min in 2% paraformaldehyde in PBS. After 3 rinses in 0.05% Triton-X100 in PBS (TBS), slides were blocked in 5% nonfat dry milk in TBS for 1 h at 37 °C and incubated overnight at 4 °C with mouse monoclonal anti-γ-H2AX antibody (Upstate Biotechnology, Lake Placid, NY, USA) diluted 1:1000 in 10% goat serum, 5% nonfat dry milk in TBS. Slides were then washed 3 times in PBS, blocked in 5% nonfat dry milk in PBS for 30 min at 37 °C and finally incubated for 2 h at 37 °C with Alexa 488-conjugated goat antimouse IgG (Molecular Probes, Eugene OR, USA) diluted 1:2000 in 10% goat serum, 5% nonfat dry milk in PBS. Slides were rinsed in PBS and mounted in Vectashield mounting medium with DAPI (Vector Laboratories Inc., Burlingame, CA, USA).

Slides were viewed under 1000× magnification using an Olympus fluorescence microscope (Olymphus Optical co. Tokyo, Japan) equipped with CCD camera. Round spermatids were identified by their morphology according to Mahadevaiah *et al*. [41]. At 0.5 and 2 h post-irradiation time points there were tens of small foci on multiple focal planes and it was not possible to reliably count their number. For this reason, spermatids were simply classified in γ-H2AX positive and γ-H2AX negative (Figure 6). At 48 h after irradiation, few large countable foci were observed in positive cells, and their average number was also determined. At least 100 spermatids were analyzed per mouse.

### 3.5. Flow Cytometric DNA Content Analysis of Testis Cells

Flow cytometric (FCM) DNA content analysis was performed 48 h after irradiation on one testis of control and irradiated mice to evaluate radiation-induced cytotoxicity. The procedure used is described in detail elsewhere [57]. The DNA content of the testis cells was measured using a FACScan flow cytometer (Becton Dickinson, San Jose, CA, USA). A total of 1 × 10^4^ events were accumulated for each measurement. Typical DNA content fluorescence intensity distribution histograms from adult mouse testicular cells (Figure 3B) are characterized by four main peaks representing elongated haploid spermatids, round haploid spermatids, cells with a 2C DNA content including G1 somatic and germ cells plus secondary spermatocytes, and cells with a 4C DNA content including G2 somatic and germ cells and primary spermatocytes; S-phase cells are included between 2C and 4C cell compartments, as described in Zante *et al*. [58]. The calculation of the relative frequencies of the various testicular cell types was performed automatically according to the model described in Lampariello and coworkers [59].

### 3.6. Statistical Analysis

Individual mouse data were considered the experimental unit. Mean values and standard errors relative to each homogeneous group were calculated. Statistical analysis was performed using STATISTICA software (StatSoft, Inc., Tulsa, OK, USA). Comparison between group means was performed by one-way ANOVA, and Duncan’s test was used for *post hoc* comparisons. Differences were considered due to the treatment when their probability level was lower than 5%.

## 4. Conclusions

In summary, our results suggest that in round spermatids lack of PARP1 delays the repair of radiation-induced DNA damage, whereas it does not seem to affect H2AX phosphorylation dynamics signaling double strand breaks or post-repair chromatin modifications. Additional experiments could clarify if these conclusions could be extended also to the radiation response of round spermatids in the low-dose range.

## Figures and Tables

**Table 1:** Percentage of testis cells in each population (standard error) as evaluated by flow cytometric DNA content analysis.

	Elongated spermatids	Round spermatids	Diploid cells	S-phase cells	Tetraploid cells
WT control	23.11 (1.06)	49.85 (1.13)	11.32 (0.52)	2.69 (0.13)	10.90 (0.20)
*PARP1^−^^/^^−^* control	23.71 (0.31)	48.42 (0.66)	11.36 (0.31)	2.46 (0.16)	11.07 (0.27)
WT 4 Gy 48 h	23.21 (0.61)	49.32 (0.93)	12.13 (0.91)	1.04 (0.22) b	10.94 (0.33)
*PARP1^−^^/^^−^* 4 Gy 48 h	26.28 (0.79) a	46.83 (0.93)	11.57 (0.54)	0.70 (0.11) b	11.29 (0.27)

a *p* < 0.05;

b *p* < 0.005.

**Table 2:** Mean fraction tail DNA (± standard error) of unirradiated testis cells and of 4 Gy-irradiated cells sampled immediately, 2 or 48 h after exposure.

	WT Fraction tail DNA (± s.e.)	*PARP1^−/−^* Fraction tail DNA (± s.e.)
Control	0.09 (0.01)	0.08 (0.01)
4 Gy T0	0.14 (0.02) a	0.13 (0.01) a
4 Gy T2	0.08 (0.01)	0.11 (0.01) a
4 Gy T48	0.09 (0.01)	0.08 (0.01)

a *p* < 0.05 with respect to matched controls.
